# Plumbagin Elicits Cell-Specific Cytotoxic Effects and Metabolic Responses in Melanoma Cells

**DOI:** 10.3390/pharmaceutics13050706

**Published:** 2021-05-12

**Authors:** Haoran Zhang, Aijun Zhang, Anisha A. Gupte, Dale J. Hamilton

**Affiliations:** 1 Center for Bioenergetics, Houston Methodist Research Institute, Houston, TX 77030, USA; hzhang@houstonmethodist.org (H.Z.); azhang@houstonmethodist.org (A.Z.); aagupte@houstonmethodist.org (A.A.G.); 2 Department of Dermatology, Xiangya Hospital, Central South University, Changsha 410008, China; 3 Molecular Biology Research in Medicine, Houston Methodist Research Institute, Weill Cornell Medicine Affiliate, Houston, TX 77030, USA; 4 Department of Medicine, Houston Methodist, Weill Cornell Medicine Affiliate, Houston, TX 77030, USA

**Keywords:** melanoma, plumbagin, cytotoxic effect, metabolism, mitochondria, reactive oxygen species

## Abstract

Melanoma is one of the most malignant skin cancers that require comprehensive therapies, including chemotherapy. A plant-derived drug, plumbagin (PLB), exhibits an anticancer property in several cancers. We compared the cytotoxic and metabolic roles of PLB in A375 and SK-MEL-28 cells, each with different aggressiveness. In our results, they were observed to have distinctive mitochondrial respiratory functions. The primary reactive oxygen species (ROS) source of A375 can be robustly attenuated by cell membrane permeabilization. A375 cell viability and proliferation, migration, and apoptosis induction are more sensitive to PLB treatment. PLB induced metabolic alternations in SK-MEL-28 cells, which included increasing mitochondrial oxidative phosphorylation (OXPHOS), mitochondrial ATP production, and mitochondrial mass. Decreasing mitochondrial OXPHOS and total ATP production with elevated mitochondrial membrane potential (MMP) were observed in PLB-induced A375 cells. PLB also induced ROS production and increased proton leak and non-mitochondria respiration in both cells. This study reveals the relationship between metabolism and cytotoxic effects of PLB in melanoma. PLB displays stronger cytotoxic effects on A375 cells, which exhibit lower respiratory function than SK-MEL-28 cells with higher respiratory function, and triggers cell-specific metabolic changes in accordance with its cytotoxic effects. These findings indicate that PLB might serve as a promising anticancer drug, targeting metabolism.

## 1. Introduction

Cutaneous melanoma, mainly caused by exposure to ultraviolet light, is the most dangerous skin cancer. It accounts for 1% of skin cancer diagnoses but results in 90% of deaths from all skin cancers [1]. Surgery is generally considered a typical treatment for localized melanoma. Multidisciplinary approaches, including chemotherapy, immunotherapy, and radiation therapy, are performed in advanced cases to improve the prognosis since advanced melanomas display high aggressiveness and a metastatic characteristic [2]. With current evidence-based treatment, cutaneous melanoma with lymph node and distant metastasis is reported to have 66.2% and 27.3% five-year survival rates, respectively, in the United States compared with up to 99% survival for localized melanoma [3]. Accordingly, it is necessary to explore novel treatments in the management of melanomas with different aggressiveness. A375 and SK-MEL-28 are two human melanoma cell lines with the BRAF mutation at V600E; they were selected as representative models according to the Melanoma Aggressiveness Score (MAGS). A375 has a higher MAGS than SK-MEL-28 because of the great differences in cell proliferation, migration, invasion, cell-doubling time, and other aggressive phenotypes [4].

Several natural phytochemical compounds with high anticancer potential are promising drugs in cancer therapy, including the treatment of melanoma [5,6,7]. Plumbagin (PLB), a naphthoquinone derivative mainly extracted from the plant, exhibits anticancer potential in different cancers, including inhibition of invasion and metastasis, induction of autophagy and apoptosis, and alteration of the cell cycle [8,9,10,11]. PLB is also considered a potent suppressor of cellular glutathione and an inducer of ROS [9,12]. Furthermore, PLB shows an anticancer effect on breast cancer depending on NQ01 activity [13], an inhibiting effect on mitochondrial electron transport, and an Nrf-2-mediated antioxidative response in several cancer cell lines [14]. It indicates the latent role of PLB on energy metabolism and mitochondrial bioenergetics. PLB has been proven to have anti-invasion and anti-metastasis effects via MAPK pathways [15] and the effect of triggering mitochondrial apoptosis by ROS and c-Jun N-terminal kinase pathways in melanoma cells [12].

However, little is known about the metabolic effect of PLB on human melanoma cells with different aggressiveness. This study aims to investigate the phenotypic alteration of cancer progression and energy metabolic changes in melanoma cell lines with different aggressiveness by applying the PLB treatment in vitro.

## 2. Materials and Methods

### 2.1. Chemicals and Reagents

Plumbagin from Plumbago indica (P7262), superoxide dismutase (SOD, S8409), L-glutamic acid (49449), L-malic (M1000), adenosine 5′-diphosphate monopotassium salt dihydrate (A5285), pyruvate (P2256), succinate (S2378), digitonin (D5628), oligomycin (O4876), carbonyl cyanide-4-(trifluoromethoxy) phenylhydrazone (FCCP, C2920), rotenone (R8875), antimycin A (A8674), L-glutamine solution (200mM, 59202C), D-(+)-glucose (G7021), H_2_O_2_ solution, 323381), and propidium iodide (PI, 11348639001) were purchased from Sigma (St. Louis, MO, USA). Dulbecco’s modified Eagle’s medium (DMEM, 30-2002), Eagle’s minimum essential medium (EMEM, 30-2003), dermal cell basal medium (PCS-200-030), and the Adult Melanocyte Growth Kit (PCS-200-042) were obtained from American Type Culture Collection (ATCC, Manassas, VA, USA). Sodium pyruvate solution (1136-070) was obtained from Gibco (Grand Island, NY, USA). Horseradish peroxidase (A22188), Amplex UltraRed (AmR, A36006), MitoTracker Green dye (M7514), and Hoechst 33,258 (H3569) were purchased from Invitrogen (Foster City, CA, USA). Total OXPHOS human WB antibody cocktail (MS601) was purchased from Abcam (Cambridge, UK). GAPDH rabbit antibody (2118) was purchased from Cell Signaling Technology (Danvers, MA, USA). Goat anti-rabbit IgG-HRP (sc-2004) and goat anti-mouse IgG-HRP (sc-2005) were obtained from Santa Cruz BioTechnology (Dallas, TX, USA); 4–20% protein gels (456-1096) were purchased from BioRad (Hercules, CA, USA). A nonradioactive cell proliferation MTS assay (G6521) was purchased from Promega (Madison, WI, USA). XF DMEM base medium (102353-100), a Seahorse XF cell Mito Stress Test kit (103015-100), and a Seahorse XF Real-Time ATP Rate Assay kit (103592-100) were obtained from Agilent (Santa Clara, CA, USA). A MiR05 kit (60101-01) was purchased from Oroboros (Innsbruck, Austria). Annexin V-FITC (556419) and 10X Annexin V binding buffer (556454) were purchased from BD Biosciences (San Jose, CA, USA).

### 2.2. Cell Culture

Human melanoma cell lines A375 (ATCC-CRL-1619), with higher aggressiveness, SK-MEL-28 (ATCC-HTB-72), with lower aggressiveness, and normal human primary epidermal melanocytes (HEMa, ATCC-PCS-200-013) were purchased from ATCC. A375 cells were cultured in DMEM and SK-MEL-28 cells were cultured in EMEM; both kinds of the medium were supplemented with 10% fetal bovine serum, 100 IU/mL penicillin, and 100 µg/mL streptomycin. HEMa cells were cultured in dermal cell basal medium supplemented with an Adult Melanocyte Growth Kit. Cells were maintained at 37 °C in a humidified incubator with 5% CO_2_. A 0.25% (*w/v*) trypsin-0.53mM EDTA solution was used to detach and collect the cells for subculturing or further experiments.

### 2.3. Dose–Response Curve Analysis

PLB was dissolved in DMSO at a series of stock concentrations and 1:1000 diluted to specific concentrations for cell treatments. The control group had the same volume of DMSO added during the treatment. A375 and SK-MEL-28 cells were seeded in a 96-well plate at the density of 1 × 10^4^ cells/well, A375 cells were treated with DMSO and PLB with concentrations of 1, 2, 2.5, 3, 3.5, 4, 6 µM, while DMSO and 1, 2, 4, 6, 7, 8, and 10 µM PLB were added to the SK-MEL-28 wells four times to repeat for each condition. HEMa cells were seeded at 5 × 10^4^ and treated with DMSO and 1, 2, 4, 6, 7, 8 µM PLB to test its cytotoxic effect on normal cells. An MTS assay was applied to assess the number of viable cells after 48 h incubation with different concentrations of PLB. Briefly, 20 ul of MTS solution was added to each well with 100 µL of renewed medium. After 2 h incubation at 37 °C in a humidified, 5% CO_2_ atmosphere, the absorbance was measured at 490 nm using a microplate reader (BioTek Instruments, Winooski, VT, USA). The IC50 values were calculated by GraphPad Prism 8.1.0 (GraphPad Software Inc., San Diego, CA, USA).

A375 and SK-MEL-28 cells were seeded at the density of 6 × 10^4^ and 4 × 10^4^ cells/well, respectively, in the 12-well plate and treated with DMSO and 1 and 3 µM PLB. Each condition was repeated twice. Trypan blue was used to determine viable cells. The cell numbers were counted at the same time each day within the following 4 days.

### 2.4. Determination of Cell Migration

Cell migration was measured by the wound-healing method. After two cell lines reaching confluence in 6-well culture plates, sterile P-1000 pipette tips were used to make artificial parallel scratches on the cell layer, and the floating cells were washed twice with PBS, which was finally replaced with complete culture medium containing DMSO or 1 or 3 µM PLB. The distance of migration was photographed with a microscope at different time points and quantified with ImageJ software (US National Institutes of Health, Bethesda, MA, USA), and the migration inhibition rate was calculated by the formula below:

$$migration\;inhibition\;rate=\frac{distance\;of\;PLB-distance\;of\;DMSO}{distance\;of\;initial\;scratch}$$

### 2.5. Apoptosis Analysis

The percentage of apoptotic A375 and SK-MEL-28 was quantified by Annexin V-FITC/PI double-staining. Briefly, cells were pretreated with varying doses of PLB (control, 1 and 3 µM) for 48 h. Harvested cells were resuspended in 100 µL 1X binding buffer and labeled for 15 min with 5 µL Annexin V-FITC and 1 µL of the 50 µg/mL PI at room temperature in the dark. A positive control group was conducted by heating cells at 55 °C for 16 min. The green (FITC) and red (PE-Texas Red) fluorescence were evaluated by flow cytometric analysis (FACSAria™ II, BD Biosciences, San Jose, CA, USA).

### 2.6. Seahorse Real-Time Cell Metabolic Analysis

Two melanoma cell lines were seeded in seahorse XF96 cell culture microplates at the density of 20,000 per well the day before the experiment. After 24 h of incubation, cells were rinsed and replaced with 180 μL of XF DMEM base medium supplemented with 1 mM pyruvate, 2 mM glutamine, and 25 mM glucose for A375 or 5.56 mM glucose for SK-MEL-28, with pH adjusted to 7.4, and incubated in a 37 °C, non-CO2 incubator for at least 1 h. The cellular oxygen consumption rate (OCR) and the extracellular acidification rate (ECAR) were measured by the Seahorse XF96e extracellular flux analyzer (Agilent, Santa Clara, CA, USA), during which the Seahorse XF cell Mito Stress Test Kit, consisting of oligomycin (Oligo), FCCP, and a mixture of ROT and AA, was added in turn to reach 1, 2, 0.5, and 0.5 μM final concentration; specifically, DMSO and 1, 3, and 6 μM PLB were injected acutely prior to the Oligo in control and PLB treatment groups. The ATP assay with acute injection consisted of DMSO or 1, 3, or 6 μM PLB, 1 μM Oligo, and the combination of 0.5 μM ROT and 0.5 μM AA. Each sample was run in triplicates at least. An Agilent Seahorse XF Mito Stress Test Report Generator (Agilent, Santa Clara, CA, USA) was applied to data analysis.

### 2.7. High-Resolution Respirometry and ROS Production Analysis

Cells were exposed to the DMSO or PLB treatment the day prior to the experiment, then harvested and resuspended with complete culture medium or MiR05 buffer at pH 7.4. The oxygen consumption and H_2_O_2_ production rates were measured with a high-resolution Oroboros respirometer (Oroboros Instruments Corp., Innsbruck, Austria). After calibration with H_2_O_2_ solution, each of the two chambers of the Oroboros machine was filled with 2 mL of the cell suspension along with SOD (2 µL, 5 kU/mL), HRP (4 µL, 500 U/mL), and AmR (2 µL, 10 mM). After 5 to 10 min stabilization, the oxygen consumption rate (OCR) and the H_2_O_2_ production rate were monitored dynamically by series of injections with substrates and inhibitors, the order of which was as the following: L-glutamate (10 µL, 2 M), malate (5 µL, 0.8 M), ADP (10 µL, 0.5 M), pyruvate (5 µL, 2 M), succinate (5 µL, 1 M), digitonin (3 µL, 8.1 mM), Oligo (3 µL, 5 mM), an inhibitor of ATPase synthetase, FCCP (3 µL, 1 mM), an oxidative phosphorylation (OXPHOS) uncoupler, ROT (1 µL, 1 mM), a complex I inhibitor, and AA (1 µL, 5 mM), a complex III inhibitor. Data were normalized by cell number and analyzed by DatLab 4 software (OROBOROS Instruments Corp., Innsbruck, Austria).

### 2.8. Mitochondria Mass Assessment

A MitoTracker Green probe that was able to diffuse across the cell membrane and accumulate in active mitochondria was used to assess mitochondria mass. Hoechst and PI were used to distinguish live from dead cells. In short, cells were seeded in a 4-well chamber slide at a density of 5 × 10^4^ (A375) or 2 × 10^4^ (SK-MEL-28) with DMSO or 1 μM PLB treatment. After 24 h incubation, cells were labeled with 50 nM MitoTracker Green dye and 2 µg/mL Hoechst in PBS at 37 °C for 25 min, protected from light. After washing with PBS, cells were covered and sealed on the slide. Fluorescence imaging was performed using a fluorescence microscope (Eclipse Ti, Nikon, Tokyo, Japan), and the intensity of fluorescence was quantified by ImageJ software (version 1.53e, National Institutes of Health, Bethesda, MD, USA).

### 2.9. Measurement of Mitochondria Membrane Potential

JC-1 dye, a kind of mitochondria membrane potential probe, was applied to measure the polarization changes due to the PLB treatment. After pretreated with DMSO or 1 or 3 µM PLB for 48 h, A375 and SK-MEL-28 cells were collected and resuspended with PBS consisting of 2 µM JC-1 dye. FCCP and JC-1 dye, both with 2 µM working concentration, were applied successively to the positive control cells. All the samples were incubated in the dark at room temperature for 15 min. To assess the attached cells with treatment for 24 h, cells were processed as we described in the Mitochondria Mass Assessment section and stained with 2 µM JC-1 dye and 2 µg/mL Hoechst, with 15-min incubation in a cell culture incubator at 37 °C. Thereafter, cells were washed once with PBS before measurement. Stained cells were examined through flow cytometry and a fluorescence microscope in FITC (green) and PE Texas-Red (red) channels.

### 2.10. Western Blotting

Proteins were extracted from DMSO and 1 and 3 µM PLB pretreated melanoma cells by RIPA buffer with protease inhibitor. After measuring the protein concentration, an equal volume of protein samples with equal concentration were loaded to 4–20% protein gels and transferred onto polyvinylidene difluoride membranes. The membranes were then blocked with 5% fatty-acid-free milk and incubated with total OXPHOS human WB antibodies (1:500) at 4 °C overnight. Next, the membranes were incubated with corresponding secondary antibodies (1:2000) for 1.5 h. The chemiluminescence bands were detected by ECL Western blotting substrate. To normalize the expression of the target protein, we stripped the membrane and incubated it with the GAPDH antibody. Following the steps we described above, the control bands were obtained. The expression of target proteins was measured by ImageJ software.

### 2.11. Statistical Data Analysis

The data were analyzed by GraphPad Prism 8 and displayed as mean ± standard deviation. To compare the data between the two groups, a *t*-test was used. Analysis of variance (ANOVA) was performed in the comparison of more than two groups, followed by Tukey’s test for posthoc analysis. *p* < 0.05 was considered statistically significant.

## 3. Results

### 3.1. A375 and SK-MEL-28 Melanoma Cells Display Comparative Mitochondria Respiratory Phenotypes

**Figure 1:**
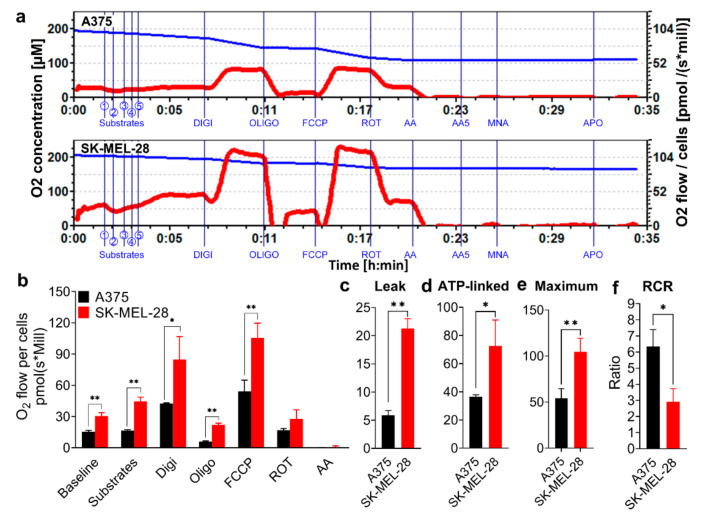
Comparative mitochondria phenotypes in melanoma cell lines. A375 and SK-MEL-28 cells were counted and placed in the Oroboros respiratory chambers, with sequential injections of Cx I and Cx II substrates (① L-glutamate; ② L-malic; ③ ADP; ④ pyruvate; ⑤ succinate), Digi (8.1 mM), Oligo (5 mM), FCCP (1 mM), ROT (1 mM), and AA (5 mM). (**a**) The O_2_ concentration (blue line) and OCR for per million A375 and SK-MEL-28 cells (red line) were monitored in real-time. (**b**) The stable OCR values for per million A375 and SK-MEL-28 cells, measured by Oroboros, are displayed in the bar graph (*n* = 3). The data accessed from Oroboros was further calculated. The results are shown as (**c**) proton leak, (**d**) ATP-linked respiration, (**e**) maximum respiration, and (**f**) RCR, which means the State 3 over State 4 oligo. Results are expressed as mean ± SD. * *p* < 0.05, ** *p* < 0.01. Cx I, complex I; Cx II, complex II; ADP, adenosine diphosphate; Digi, digitonin; Oligo, oligomycin; FCCP, carbonyl cyanide-4-(trifluoromethoxy) phenylhydrazone; ROT, rotenone; AA, antimycin A; OCR, oxygen consumption rate; ATP, adenosine triphosphate; RCR, respiratory control ratio; SD, standard deviation.

SK-MEL-28 cells displayed a stronger mitochondrial oxygen consumption rate (OCR) with the injection of different substrates and blockers compared to the A375 cells (Figure 1a–c). After using digitonin to permeabilize the cell membrane in the Oroboros machine, the basal mitochondria OCR increased in both A375 and SK-MEL-28 cells since the substrates of the mitochondrial complex I (Cx I) and complex II (Cx II), including L-glutamate, L-malic, pyruvate, and succinate, along with adenosine diphosphate (ADP), entered the cells and were oxidized by mitochondria in the mitochondria respiration buffer (MiR05; Figure 1a,b). Oligomycin (Oligo), an inhibitor of adenosine triphosphate (ATP) synthase, blocks the proton channel of complex V (Cx V). The comparative mitochondrial proton leak in SK-MEL-28 cells was higher than in A375 cells, as determined by assessing the difference of the OCR between oligomycin and antimycin A (AA) (Figure 1c). ATP-linked respiration, the value of digitonin (Digi) minus the value of Oligo, was higher in SK-MEL-28 cells (Figure 1d). FCCP, a mitochondrial OXPHOS uncoupler that facilitates the proton transfer across the mitochondrial inner membrane by bypassing the F0 subunit of the Cx V pathway, increased the OCR to the maximum in both cells. The maximum mitochondrial respiration in A375 cells was inferior to SK-MEL-28 cells (Figure 1e). The respiratory control ratio (RCR) is defined as the ratio of ADP-stimulated respiration (State 3) to leak respiration when ATP production is shut off by Oligo (State 4 oligo). The RCR of A375 cells was higher than SK-MEL-28 cells (Figure 1f), indicating a high capacity for substrate oxidation [16].

### 3.2. H_2_O_2_ Production Rates Vary in A375 and SK-MEL-28 Cells

**Figure 2:**
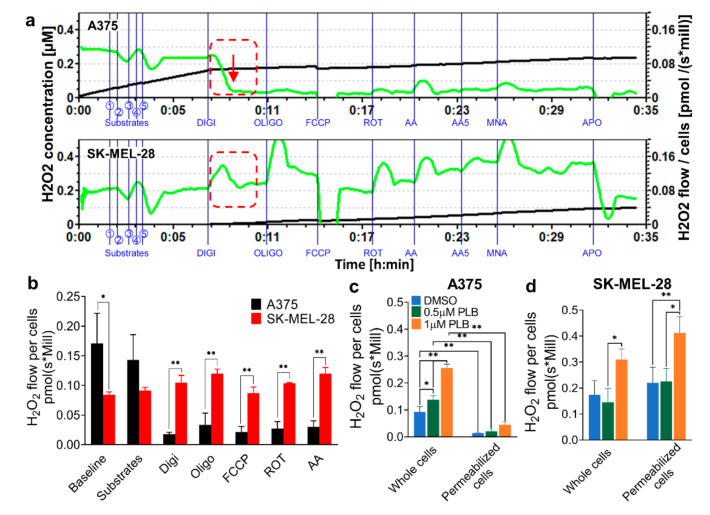
H_2_O_2_ production rate for whole and permeabilized melanoma cells at basal and PLB induced levels. A375 and SK-MEL-28 cells were counted and placed in the Oroboros respiratory chambers, with sequential injections of Cx I and Cx II substrates (① L-glutamate; ② L-malic; ③ ADP; ④ pyruvate; ⑤ succinate), Digi (8.1 mM), Oligo (5 mM), FCCP (1 mM), ROT (1 mM), and AA (5 mM). H_2_O_2_ signals were detected by Amplex UltraRed. (**a**) The H_2_O_2_ level (black line) and H_2_O_2_ flow for per million A375 and SK-MEL-28 cells (green line) were monitored in real-time. The red dashed box, along with a red arrow, highlights the H_2_O_2_ production drop in A375 cells compared to SK-MEL-28 cells. (**b**) The stable H_2_O_2_ flow values for per million A375 and SK-MEL-28 cells are displayed in the bar graph. (*n* = 3) The H_2_O_2_ production rates for whole and permeabilized (**c**) A375 cells and (**d**) SK-MEL-28 cells with DMSO and 1 and 3 µM PLB treatments are represented as the H_2_O_2_ flow before and after digitonin is applied (*n* = 3). Results are expressed as mean ± SD. * *p* < 0.05, ** *p* < 0.01. DMSO, dimethyl sulfoxide; PLB, plumbagin.

AmR fluorescence was recorded concurrently with OCR, as above (Figure 2a,b). A375 cells displayed a higher H_2_O_2_ production rate than SK-MEL-28 cells at the baseline without the addition of digitonin. Surprisingly, the H_2_O_2_ production rate dropped significantly, with no response to the addition of any blockers to A375 cells after cell membrane permeabilization occurred with the injection of digitonin. By contrast, digitonin has no effect on the H_2_O_2_ production rate in SK-MEL-28 cells, which were unresponsive to the blockers (Figure 2a,b).

### 3.3. H_2_O_2_ Production Triggered Differentially by PLB in A375 and SK-MEL-28

Melanoma cells were pretreated with DMSO and 0.5 and 1 µM PLB and subsequently injected into the chambers of Oroboros. Before digitonin application, PLB induced H_2_O_2_ production in both intact A375 and SK-MEL-28 cells. The increased H_2_O_2_ production rate in the DMSO group and PLB groups declined in A375 cells permeabilized with digitonin (Figure 2c). Conversely, the rate in permeabilized SK-MEL-28 cells slightly increased and was amplified by PLB treatment (Figure 2d).

### 3.4. Inhibitory Effects of PLB on Cell Viability and Proliferation Are Robust in A375 Cells

**Figure 3:**
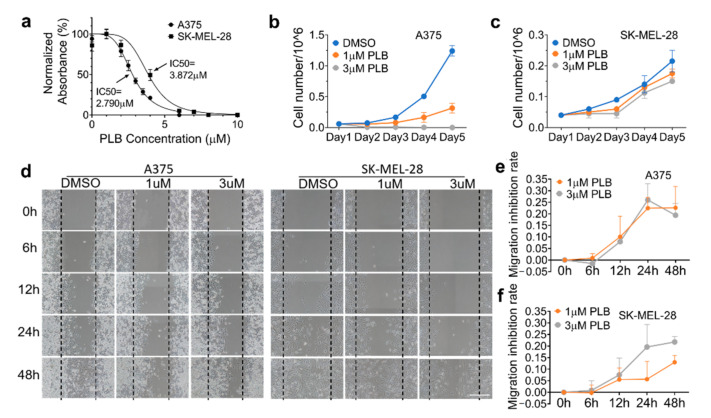
Differential inhibition effects of PLB on cell viability and migration in A375 and SK-MEL-28. (**a**) A375 and SK-MEL-28 cells were seeded in the 96-well plate at the density of 1 × 10^4^ cells/well and treated with PLB (DMSO and 1, 2, 2.5, 3, 3.5, 4, and 6 µM PLB for A375 cells; DMSO and 1, 2, 4, 6, 7, 8, and 10 µM PLB for SK-MEL-28 cells) for two days. Viable cell numbers were measured by MTS assay. The IC50 of A375 and SK-MEL-28 cells were 2.790 and 3.872 µM, respectively (*n* = 3). (**b**) A375 cells and (**c**) SK-MEL-28 cells treated with DMSO and 1 and 3 µM PLB were counted for 5 days (*n* = 2). (**d**) Two cell lines were seeded in a 6-well plate, and the migration status was displayed by different treatments (DMSO and 1 and 3 µM PLB) and time points (0, 6, 12, 24, and 48 h). The migration inhibition rates in the 1 and 3 µM PLB groups were compared with the DMSO group. Scale bar = 0.1 mm. (**e**) Migration inhibition rate of A375 cells. (**f**) Migration inhibition rate of SK-MEL-28 cells (*n* = 3). Results are expressed as mean ± SD. MTS, nonradioactive cell proliferation assay; IC50, 50% inhibitory concentration.

Metabolically active cells were able to react with the MTS assay, a number of which were positively related to the absorbance value detected by the microplate reader. The dose–response curves were displayed in Figure 3a. Based on this, the 50% inhibitory concentration (IC50) of PLB was calculated. The results revealed that PLB inhibited cell viability and proliferation in A375 cells (IC50 = 2.790 μM) more effectively than in SK-MEL-28 cells (IC50 = 3.872 μM). PLB has no cytotoxic effect on normal melanocytes (HEMa). On the contrary, we found an increasing survival rate of HEMa cells with PLB treatment, even in high concentrations, compared with the DMSO group (Figure S1). Moreover, time and dose–response of the PLB treatment were also determined by cell number counting. In the control group, A375 cells displayed a stronger exponential growth pattern than SK-MEL-28 cells over time. The growth of A375 cells was significantly inhibited by 1 µM PLB, and the inhibition effect was even stronger in the 3 µM PLB group (Figure 3b). However, PLB’s inhibitory effect on SK-MEL-28 was not as strong compared with that on A375 cells with the 1 and 3 µM PLB treatments (Figure 3c).

### 3.5. Inhibitory Effects of PLB on A375 and SK-MEL-28 Cell Migration

The migration results of two melanoma cell lines with DMSO or 1 or 3 µM PLB treatment, monitored by microscopy at different time points (0, 6, 12, 24, and 48 h), are exhibited in Figure 3d. The inhibition of migration is defined as the difference of cell migration distance between DMSO and PLB groups, which is divided by the width of scratch at the beginning for each condition, respectively. In A375 cells, PLB had a relatively parallel inhibitory effect at concentrations of 1 and 3 µM (Figure 3e), while only a 21.8% inhibition of migration was seen in SK-MEL-28 cells with the 3 µM treatment. A low concentration of PLB treatment in SK-MEL-28 cells was unable to induce a similar suppression effect as in A375 cells (Figure 3f).

### 3.6. PLB-Induced Apoptosis in A375 But Not SK-MEL-28 Cells

**Figure 4:**
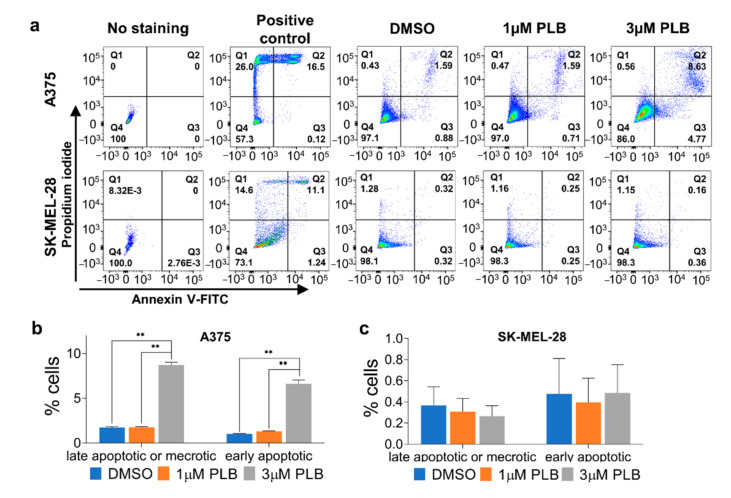
Differential effects of PLB on inducing A375 and SK-MEL-28 apoptosis. A375 and SK-MEL-28 cells pretreated with DMSO and 1 and 3 µM PLB were stained with Annexin V-FITC and PI. Cells heated at 55 °C for 16 min were the positive controls. (**a**) Cells with fluorescence were detected by flow cytometry. (**b**) The percentage of late apoptotic or necrotic and early apoptotic A375 cells in DMSO and 1 and 3 µM PLB groups. (**c**) The percentage of late apoptotic or necrotic and early apoptotic SK-MEL-28 cells in DMSO and 1 and 3 µM PLB groups. Results are expressed as mean ± SD (*n* = 3). ** *p* < 0.01. PI, propidium iodide.

According to the cells without staining, we drew the gate for flow cytometric analysis to differentiate the positive group from the negative group. Both late apoptosis or necrosis and early apoptosis were obviously induced in the 3 µM PLB group compared with the DMSO and 1 µM PLB groups in A375 cells. There was no significant difference between the DSMO group and the 1 µM group in A375 cells (Figure 4a,b). Neither 1 nor 3 µM PLB had any visible and statistical apoptotic effect on SK-MEL-28 cells (Figure 4a,c).

### 3.7. Distinct Mitochondrial Responses to PLB Treatment in A375 and SK-MEL-28 Cells

**Figure 5:**
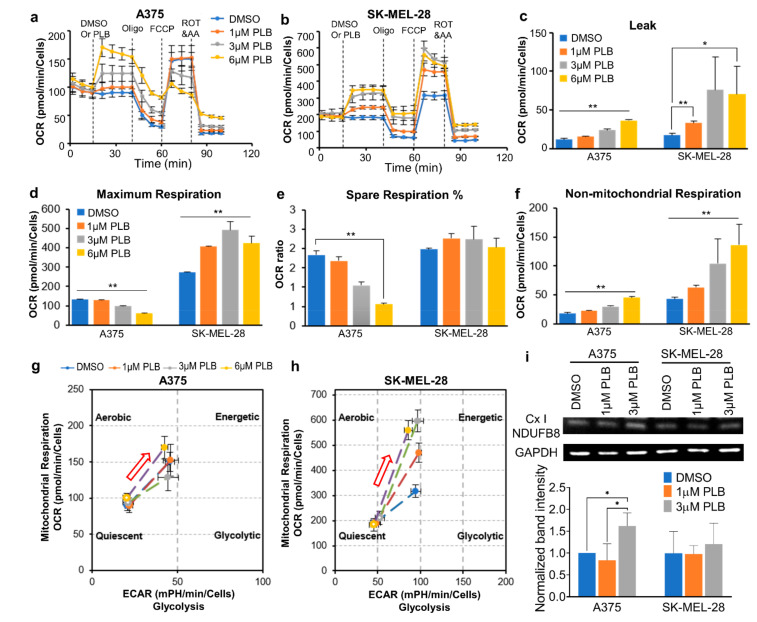
Mitochondrial respiratory function alternations due to PLB treatment. Melanoma cells were seeded in the seahorse XF96 cells culture microplates at a density of 2 × 104. DMSO and 1, 3, and 6 µM PLB were acutely applied in the Seahorse machine with the following injection of Oligo, FCCP, ROT, and AA (*n* = 3–8). (**a**) OCR of A375 cells. (**b**) OCR of SK-MEL-28 cells. (**c**) Proton leak. (**d**) Maximum respiration. (**e**) Percentage of spare respiratory capacity. (**f**) Nonmitochondrial respiration. (**g**) Energy map of A375. Red arrow shows the direction of PLB-induced energy change. (**h**) Energy map of SK-MEL-28. Red arrow shows the direction of PLB-induced energy change. (**i**) Western blot results show the expression of NDUFB8 subunit of mitochondrial Cx I with DMSO and 1 and 3 µM PLB treatment. The bar figure displays the normalized results compared with the expression of GAPDH (*n* = 3). Results are expressed as mean ± SD. * *p* < 0.05, ** *p* < 0.01. GAPDH, glyceraldehyde 3-phosphate dehydrogenase. NDUFB8, ubiquinone oxidoreductase subunit B8.

The injection of PLB (DMSO and 1, 3, and 6 µM) triggered dose-dependent increases of the basal OCR and proton leak in both melanoma cell lines (Figure 5a–c). The maximum respiratory rate in SK-MEL-28 cells directly increased with the increasing PLB doses. On the contrary, the maximum respiration decreased in the high concentration group (3, 6 µM PLB) in A375 cells (Figure 5d). Furthermore, the percentage of spare respiratory capacity declined in A375 cells (Figure 5e). Nonmitochondrial respiration was enhanced in both cell lines with increased dose titration (Figure 5f). The energy maps showed that the OCR and the extracellular acidification rate (ECAR) were induced in both cell lines with PLB. The energy metabolism in SK-MEL-28 cells transited to aerobic respiration with increasing doses of PLB, while no change was observed in A375 cells within groups (Figure 5g,h). The results from Western blotting show that the expression of the nicotinamide adenine dinucleotide hydride (NADH): ubiquinone oxidoreductase subunit B8 (NDUFB8), a major subunit supporting proton pumping in Cx I, was enhanced in the 3 µM PLB group in A375 cells. There was no difference in the expression of NDUFB8 in SK-MEL-28 cells with different doses of PLB (Figure 5i).

### 3.8. Alternation of ATP Production with PLB Treatment

**Figure 6:**
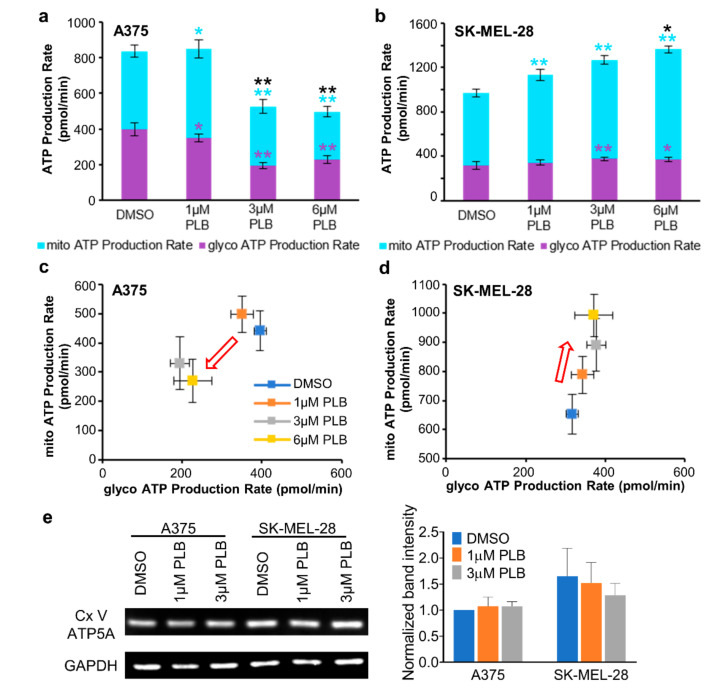
PLB induced alternations of ATP production. Melanoma cells were seeded in the seahorse XF96 cell culture microplates at the density of 2 × 104. DMSO and 1, 3, and 6 µM PLB were acutely applied in the Seahorse machine with the following injection of Oligo, ROT, and AA (*n* = 3–8). (**a**) The bar figure shows the absolute value of ATP production contributed by mitochondria and glycolysis in A375 cells. The significance for each experimental group was compared with the DMSO group. Asterisks in black, purple, and light blue represent total glycolysis and mitochondrial ATP production. (**b**) The bar figure shows the absolute value of ATP production contributed by mitochondria and glycolysis in SK-MEL-28 cells. The significance for each experimental group was compared with the DMSO group. Asterisks in black, purple, and light blue represent total glycolysis and mitochondrial ATP production. (**c**) ATP production map in A375 cells. The red arrow represents the trend of ATP production with the increasing concentrations of PLB. (**d**) ATP production map in SK-MEL-28 cells. The red arrow represents the trend of ATP production with the increasing concentrations of PLB. (**e**) Western blot result shows the expression of ATP5A, a subunit of mitochondrial Cx V in two cell lines with DMSO and 1 and 3 µM PLB treatment. The bar figure displays the normalized results compared with the expression of GAPDH (*n* = 3). Results are expressed as mean ± SD. * *p* < 0.05, ** *p* < 0.01. ATP5A, ATP synthase subunit alpha; Cx V, complex V.

Total ATP production in A375 cells decreased in the 3 and 6 µM PLB groups but increased in SK-MEL-28 cells with 1, 3, and 6 µM PLB when compared with the DMSO group (Figure 6a,b). The energy map for the ATP production rate quantitated the proportion of mitochondria-derived ATP and glycolysis-derived ATP from total ATP. For A375 cells, the points representing the 3 and 6 µM PLB-treated groups moved towards the lower-left corner, which indicated that both the mitochondrial ATP production rate and the glycolytic ATP production rate slowed in parallel (Figure 6a,c). As for SK-MEL-28 cells, the boosted total ATP production rate was mainly attributed to the dose-dependent increase of the mitochondrial ATP production rate (Figure 6b,d). However, no significant alternation for the expression of ATP synthase subunit alpha (ATP5A) in the 1 and 3 µM PLB groups was observed in Western blot data in comparison to the control group in the two cell lines (Figure 6e).

### 3.9. Effects of PLB on the Mitochondria Mass and Mitochondrial Membrane Potential (MMP)

**Figure 7:**
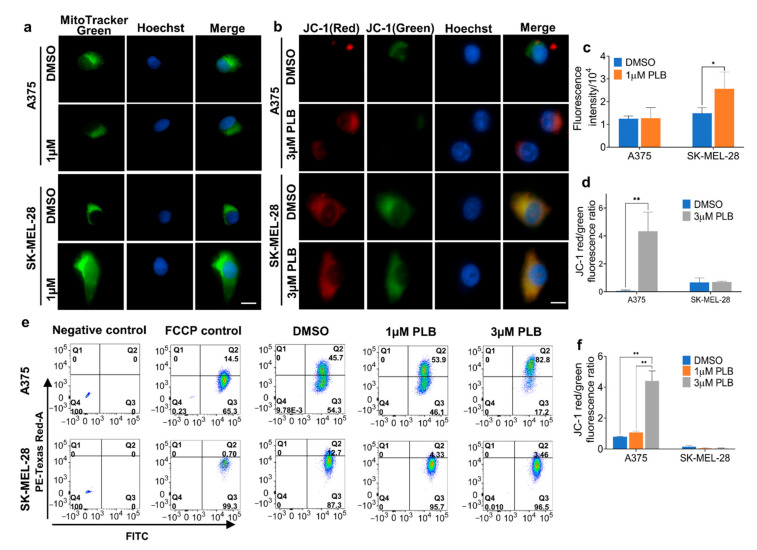
Mitochondrial mass and membrane potential. (**a**) Melanoma cells were seeded in the 4-well chambers with DMSO and 1 µM PLB, treated, and stained by 50 nM Mitotracker Green and 2 µg/mL Hoechst dye. The figure set displays the fluorescence images under a 60X microscope. Scale bar = 10 µm. (**b**) Melanoma cells were seeded in the 4-well chambers with DMSO and 3 µM PLB, treated, and stained by 2 µM JC-1 and 2 µg/mL Hoechst dye. The figure set displays the fluorescence images under a 60X microscope. Scale bar = 10 µm. (**c**) The intensity of Mitotracker Green. (**d**) The ratio of red to green intensity of JC-1. (**e**) Melanoma cells with DMSO and 1 and 3 µM PLB treatment were stained with 2 µM JC-1 and 2 µg/mL Hoechst dye and detected by flow cytometry. No staining and FCCP-treated groups were negative and positive control. (**f**) The ratio of cells with red fluorescence to cells with green fluorescence. Results are expressed as mean ± SD. * *p* < 0.05, ** *p* < 0.01.

The intensity of green fluorescence reflects the mitochondria mass, which was significantly augmented in SK-MEL-28 cells following the 1 µM PLB treatment. There was no difference in A375 cells under PLB treatment (Figure 7a,c). Furthermore, MMP alternation was confirmed with JC-1 staining in both fluorescence microscopy and flow cytometry. We display the ratio of red to green fluorescence, which is positively related to MMP; 3 µM PLB treatment resulted in a massive increase of MMP in A375 cells, whereas there was no difference within groups for SK-MEL-28 cells (Figure 7b,d). Similar results were found in flow cytometry: a low concentration of PLB (1 µM) was unable to trigger increasing MMP in A375 cells. However, there was a significant difference between the DMSO group and the 3 µM PLB group in A375 cells but similar MMP in SK-MEL-28 cells among the dosage groups (Figure 7e,f).

## 4. Discussion

Drug treatment is widely used in the treatment of malignant melanoma, the most fatal form of skin cancer, to improve the survival rate after surgery, especially in melanomas with high aggressiveness [17]. Since some melanomas with the BRAFV600E mutation are prone to become resistant to targeted inhibitors and therapeutic chemicals [18], new targets and substances are required in melanoma treatment. A plant-derived small molecular compound, PLB, was reported to have latent anticancer properties by carrying out cytotoxicity, inducing ROS production, and altering metabolism-related pathways in multiple cancer types, including melanoma [9,12,14]. This study further explored the cytotoxic and metabolic roles of PLB played in two melanoma cell lines with different aggressiveness and metabolic phenotypes.

Although both A375 and SK-MEL-28 cells harbor the BRAF mutation at V600E, they exhibit distinct aggressive biological natures, with high aggressiveness in A375 cells and low levels of aggressiveness in SK-MEL-28 cells [4]. BRAFV600F-mutated melanoma cells are associated with upregulated aerobic glycolysis and suppressed OXPHOS [19,20]. However, in line with the differences in aggressiveness, these two cell lines display different metabolic phenotypes. The results of our study document enhanced mitochondrial oxygen consumption in SK-MEL-28 cells compared with A375 cells, which harbor a more efficient basal and maximum mitochondria respiratory rate linked to ATP respiration. Proton leak respiration, which represents decreased efficiency, is higher in SK-MEL-28 cells. Previous research has also documented a relationship between high mitochondrial activity and indolent tumor behavior [21,22]. In contrast, regarding the relative OCR percentage due to ATP synthesis, as compared to proton leak, A375 cells exhibit more ATP-linked respiration and less proton leak, as indicated by the higher RCR in A375. This emerging evidence suggests that A375 cells with high aggressiveness have more advanced mitochondrial efficiency than SK-MEL-28 cells despite the lower OCR, which may contribute to cancer cell survival under hypoxia. We can speculate that these metabolic differences in two melanoma cell lines may cause variations in response to some cancer treatments.

The cytotoxic effects of PLB are proven in several cancer types, such as inhibiting proliferation and invasion and inducing apoptosis and cell cycle arrest [8,9,10]. We identified that the cytotoxic effects of PLB in A375 and SK-MEL-28 cells are consistent with previous studies. Surprisingly, the cytotoxic anticancer effects exhibit significant distinctions within these two melanoma cells in cell viability and proliferation, migration, and apoptosis. Based on the IC50 value in A375 cells and SK-MEL-28 cells, we find that PLB has more potent inhibition effects on cell viability and proliferation in the more aggressive A375 cells. The inhibitory rate of migration in SK-MEL-28 cells with low concentration (1 µM) of PLB treatment is inferior to the rate in A375 cells and high-concentration (3 µM)-treated SK-MEL-28 cells. Furthermore, 3 µM PLB can accelerate the transformation of a fair percentage of living A375 cells into early and late apoptotic cells, but not in SK-MEL-28 cells. Therefore, the distinctive cytotoxic anticancer effects of PLB in melanoma cells with different aggressiveness and metabolic phenotypes imply a potent metabolic-targeted role of PLB treatment.

Recent findings suggest that drug anticancer sensitivity can be affected by the altered metabolism of cancer cells. For one thing, differential metabolic phenotypes in cancer cells can positively impact the anticancer effects of some drugs. Therapies combined fasting-mimicking diet and other anticancer medicines, for example, metformin, have synergistic cytotoxic effects as a result of the metabolic alteration caused by fasting-mimicking diets [23,24]. For another, there is evidence proving metabolic-related drug resistance [25]. In BRAFV600E-mutated melanoma cells, upregulated OXPHOS and mitochondrial respiration, increased ROS levels, and attenuated glucose metabolism are associated with drug resistance to BRAF inhibitors [19,20,26]. Our results represent distinct metabolic profiles as well as different sensitivities to PLB in A375 and SK-MEL-28 cells: SK-MEL-28 cells, with an increased mitochondrial OCR, display low cytotoxic response to PLB. A375 cells are exactly the opposite, which implies the cell-specific and metabolic-specific anticancer sensitivity of PLB.

To expand the relationship between PLB and these two melanoma cells a little further, we investigated the metabolic alteration in PLB-treated A375 and SK-MEL-28 cells. PLB increases the respiratory rate in SK-MEL-28 cells mainly by inducing mitochondrial respiration, yet no obvious response was found in the mitostress energy map of A375 cells. Nevertheless, the increase of proton leak and decrease of maximum respiration, as well as spared respiration in A375 cells with PLB treatment, implies the negative effects of PLB in A375 cells. In SK-MEL-28 cells, the effects are positive despite the increase of proton leak because of the increased maximum respiration and stabilized spared respiration. The spared respiration, also called reserve respiration, is decreased by oxidative stress, the depletion of which, if exceeding the threshold of the basal respiration, can cause cell death [27,28]. Furthermore, we surprisingly found that mitochondria-independent respiration was stimulated by PLB in both cells. Nonmitochondrial respiration is generated from several pathways, including NADPH oxidases and lipoxygenases [27]. An elevated ROS level is also responsible for nonrespiration [29]. In our study, we first evaluated the innate abilities for ROS (H_2_O_2_) production, which are higher in A375 cells. Interestingly, after permeabilization, the ROS production rate declined sharply in A375 cells and increased a little bit in SK-MEL-28 cells. The results reveal that the major source of ROS in A375 cells may be outside the mitochondria, not mainly from the electron transport chain. Then, we confirmed that PLB induced ROS production, which is consistent with previous cancer research [9,10,11]. The reduction of ROS production due to cell permeabilization was enhanced by PLB in A375 cells. We can speculate that some oxidative enzymes, for example, the NADPH enzyme [30], may be involved in PLB-induced ROS production in A375 cells. ROS production in SK-MEL-28 cells is more dependent on mitochondrial respiration.

Our study demonstrates that PLB reduced ATP synthesis in A375 cells from both mitochondria and glycolysis, whereas mitochondrial ATP production in SK-MEL-28 cells was accelerated by PLB in a dose-dependent manner, which implies a degree of consistency with the results above. However, the expression of ATP5A basically remains stable, with or without PLB, in the two cell lines. As we described above, PLB-treated A375 cells display attenuated mitochondrial respiration; hence, more heat is generated, caused by leak-related respiration compared with ATP production [31]. Therefore, mitochondrial ATP production does not depend on the expression of ATP5A. Interestingly, we observed that MMP in A375 cells exceedingly increases with a lethal dose (3 µM) of PLB treatment. No significant MMP change was recorded in the SK-MEL-28 cells. Previous studies suggest a negative correlation between MMP and ATP production [32]. Consistently, the increase in MMP, accompanying diminished ATP production in A375 cells, is also proven in our experiments. Such an increase is reported in apoptosis and cancer [33,34]. The NDUFB8 subunit, located in the transmembrane domain of mitochondrial Cx I, functions as the proton pump to elevate MMP [35]. In our Western blotting results, the expression of NDUFB8 significantly increased in A375 cells under the 3 µM PLB treatment, which can be another explanation for the increased MMP. In addition, mitochondrial intensity is strengthened by PLB in SK-MEL-28 cells and unchanged in A375 cells. The results indicate an increased mitochondrial mass [31,36] and provide support for enhanced mitochondrial OXPHOS and bioenergetic capacity in the PLB-treated SK-MEL-28 cells we discussed above [29,37]. Based on our data and other research, PLB may also cause mitochondrial biogenesis to increase or mitophagy to decrease [38].

This investigation of melanoma cells, with different levels of aggressiveness and metabolic phenotypes, documents differential cytotoxic and metabolic responses when exposed to plumbagin. The study provides initial evidence that plumbagin, acting as a REDOX altering agent, might be utilized as an adjunct to melanoma therapy. Further studies on the anticancer characteristic of PLB could focus on specific molecular pathways, related gene expressions, and drug combination therapies.

## Data Availability

Data are contained within the article.

## Supplementary Materials

The following are available online at https://www.mdpi.com/article/10.3390/pharmaceutics13050706/s1, Figure S1: Survival rate in HEMa cells with PLB treatment.
