# Supplementary material for: Plumbagin Elicits Cell-Specific Cytotoxic Effects and Metabolic Responses in Melanoma Cells
Source: Pharmaceutics. 2021 May 12;13(5):706. doi: 10.3390/pharmaceutics13050706 (PMC8151164; doi:10.3390/pharmaceutics13050706)
Supplement: Supplementary file 1 [file pharmaceutics-13-00706-s001.zip › pharmaceutics-1165119-supplementary.pdf]

pharmaceutics

MDPI

Supplementary Materials: Plumbagin Elicits Cell-Specific Cytotoxic Effects and Metabolic Responses in Melanoma Cells

Haoran Zhang, Aijun Zhang, Anisha A. Gupte and Dale J. Hamilton

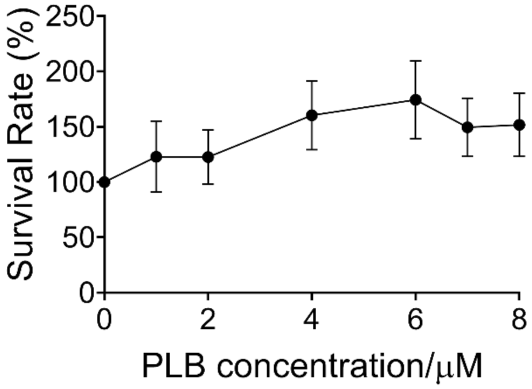

Figure S1. Survival rate in HEMa cells with PLB treatment. HEMa cells were seeded in the 96-well plate at the density of $5x10^4$ cells/well and treated with DMSO, 1, 2, 4, 6, 7, 8μM PLB for two days. Viable cell numbers were measured by MTS assay, which were normalized by the DMSO group and displayed as survival rate (%). Results are expressed as mean ± SD. HEMa, normal human primary epidermal melanocytes.
